# Neoadjuvant chemotherapy followed by surgery versus upfront surgery in non-metastatic non-small cell lung cancer: systematic review and meta-analysis of randomized controlled trials

**DOI:** 10.18632/oncotarget.20044

**Published:** 2017-08-08

**Authors:** Xiao-Nan Zhang, Lei Huang

**Affiliations:** ^1^ Department of General Surgery, The First Affiliated Hospital of Anhui Medical University, Hefei, China; ^2^ Department of Respiratory Medicine, The First Affiliated Hospital of Anhui Medical University, Hefei, China

**Keywords:** neoadjuvant chemotherapy, surgery, non-small cell lung cancer, efficacy, safety

## Abstract

**Background:**

The favorable effect of postoperative chemotherapy on long-term survival has been well acknowledged in non-small cell lung cancer (NSCLC), while the role of neoadjuvant chemotherapy (NAC) remains obscure. This meta-analysis enrolling high-quality randomized controlled trials (RCTs) aimed at comparing NAC followed by surgery with upfront surgery (US) in efficacy and safety among non-metastatic NSCLC patients.

**Materials and Methods:**

Relevant literatures were searched systematically from MEDLINE, EMBASE, and the Cochrane Library. We also screened references of relevant publications and conference proceedings. Primary outcomes were overall survival (OS), disease free survival (DFS), 3-year and 5-year survival rates, mortality, and recurrence. Secondary outcomes included tumor-free (R0) resection rates, response rate, and postoperative complications. Subgroup analysis according to ethnicity was further conducted.

**Results:**

A total of 11 eligible RCTs comparing NAC (*n* = 1624) with US (*n* = 1639) and published from 1998 to 2013 were included. Compared to US, NAC contributed to longer OS and DFS, higher 3-year and 5-year DFS rates, and lower incidences of total mortality, overall recurrence and metastasis, and tended to cause higher 5-year OS rates. NAC was associated with reduced risks in recurrence compared to US. Patients receiving NAC had lower surgery and resection rates, but higher R0 resection incidence among resected cases. NAC especially benefited occident patients. The overall NAC response rate was 52.1%, and NAC-related toxicity rate was 58.3%.

**Conclusion:**

NAC may provide better survival, reduced recurrence, and improved R0 resection rates among NSCLC patients who had surgery, especially in occident patients. Further studies are needed to clarify the ethnic differences.

## INTRODUCTION

Primary non-small cell lung cancer (NSCLC) is a significant global health burden presently, although tobacco control has gained some effects and certain treatment advances have emerged in past few decades. It remains one of the most common malignancies and the leading cause of cancer-related death worldwide [1]. Surgery provides the only chance of potential cure for NSCLC patients, while only about 1/5 of patients are suitable for curative resection and the postsurgical survival is extremely poor for patients with advanced-stage tumors [2]. Adjuvant chemotherapy has been applied to treat NSCLC since 1960, with its benefits on survival definitively demonstrated [3–7]. Adjuvant chemotherapy following surgically resected (Stage IB (> 4 cm)-IIIA) NSCLC is now considered the standard of care, but the neoadjuvant setting is less well established. Neoadjuvant chemotherapy (NAC), which is defined as chemotherapy applied before surgery, has been investigated by various trials [8–18] and systematic reviews [19, 20], with controversial results; some reported survival benefit with improved resection rate and micro-metastasis control. Since many of the studies included in the previous meta-analyses [19, 20] were non-randomized small-scale trials, the results were unconvincing with biases. Up till now, the role of NAC in NSCLC remains obscure in terms of important surgical and oncological aspects like tumor-free (R0) resection rate, objective response rate, toxicity, and prognosis, which would be clarified in this updated pooled-study with novel trials included.

Herein we conducted this meta-analysis to compare the efficacy and safety of NAC to those of upfront surgery (US) in NSCLC. In order to achieve high-quality results, we included only randomized controlled trials (RCTs) and performed this pooled-analysis according to the Preferred Reporting Items for Systematic reviews and Meta-Analysis (PRISMA) guidelines and based on intention-to-treat (ITT) analysis. In this meta-analysis more than 3000 patients were investigated, offering greater power and validity.

## RESULTS

### Characteristics of the selected RCTs

**Figure 1:**
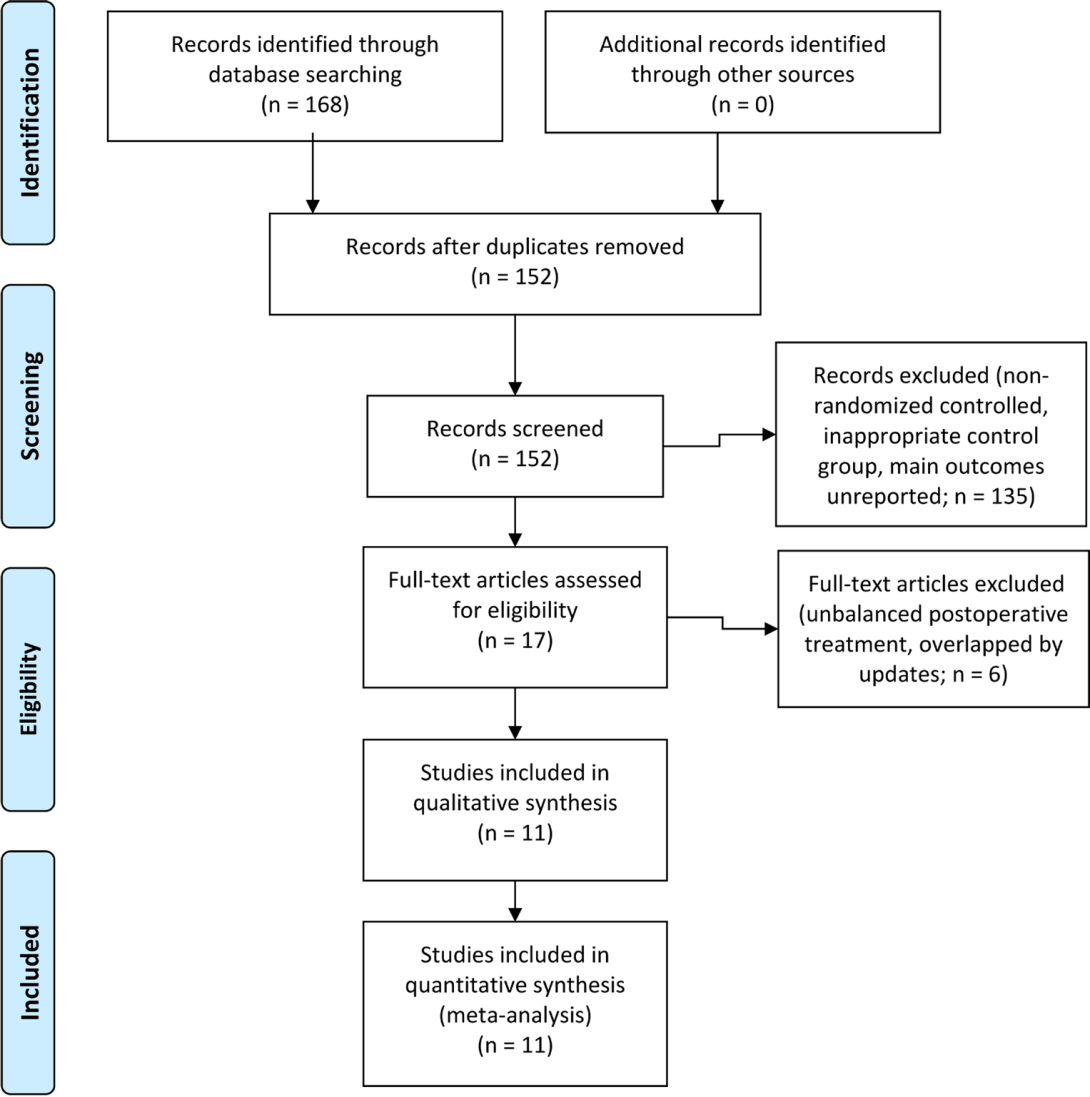
Literature selection flow diagram

A total of 168 literatures were searched out from the databases, and 69 relevant articles comparing NAC with US in NSCLC were thoroughly reviewed. According to the eligibility criteria, Dautzenberg *et al*.'s [21] and Juttner *et al*.'s [22] studies were excluded for postoperative treatment for not being strictly matched between the NAC and US groups. Rosell *et al*.'s trial in 1994 [23] had an updated assessment in 1999 [9], and Roth *et al*.’ report in 1994 [24] was renewed in 1998 [8]. Eventually, 11 studies [8–18] designed as RCTs focusing on NAC and US in the treatment of NSCLC were included (Figure 1). Among them, five trials were published from Europe, two from America, and four from Asia.

The eligible RCTs were published from 1998 to 2013, and included a total of 3263 patients with 1624 (49.8%) receiving NAC plus surgery and 1639 (50.2%) undergoing US. The information of these publications and patients’ baseline characteristics are summarized in Table 1 and Supplementary Table 1. Supplementary Table 2 shows the eligibility criteria for patient inclusion in each included trial. The NAC and US groups did not differ significantly in terms of sex (female, 20.1% *vs*. 18.8%, *P* = 0.34), Eastern Cooperative Oncology Group score (0–1, 90.8% *vs*. 92.4%, *P* = 0.79), histology type (squamous cell carcinoma [48.3% *vs*. 47.0%, *P* = 0.47]; adenocarcinoma [29.1% *vs*. 30.6%, *P* = 0.34]), tumor stage (T0–1, 4.1% vs. 4.7%, *P* = 0.63; TNM I, 37.0% vs. 39.6%, *P* = 0.24), or median follow-up duration (56.2 *vs*. 54.5 months, *P* = 0.33).

**Table 1:** Details of included trials in this meta-analysis

Authors/Trial acronym	Year, ethnicity	Accrual period	Countries where conducted		Intention to treat analysis	Matched factorsa		Sample size	Primary endpoint
Roth *et al*. [4]	1998, American	1987–1993	America (multi-center)		YES	1, 2, 4, 8		60	OS
Rosell *et al*. [5]	1999, Spanish	1989–1991	Spain (multi-center)		YES	1–5		60	OS, DFS
Zhou *et al*. [6]	2001, Chinese	1990–2001	China (multi-center)		YES	1, 2, 4, 5, 6, 8		624	OS
Depierre *et al*. [7]	2002, French	1991–1997	France (multi-center)		YES	1–4, 6		355	OS
Liao *et al*. [8]	2003, Chinese	1995–1997	China (multi-center)		YES	1, 2, 4, 8		211	OS
JCOG [9]	2003, Japanese	1993–1998	Japan (multi-center)		NR	1, 2, 5, 8		62	OS, DFS
Gilligan *et al*. [10]	2007, European	1997–2005	Europe (multi-center)		YES	1–4, 8		519	OS
Felip *et al*. [11]	2010, European	2000–2007	Europe (multi-center)		YES	1–4, 8		409	OS, DFS
Pisters *et al*. [12]	2010, American	1999–2004	America (multi-center)		YES	1–4, 8		337	OS, DFS
Scagliotti *et al*. [13]	2012, European	2000–2004	Europe (multi-center)		YES	1, 2, 4, 8		270	OS, DFS
Chen *et al*. [14]	2013, Chinese	1995–2001	China (multi-center)		NO	1, 2, 4, 8		356	OS
**Authors/Trial acronym**	**Main inclusion criteria**			**Regimen and administration**			**Follow–up duration(months)**		
Roth *et al*. [4]	Resectable NSCLC, stageIIIA, M0, Zubrod perform status 0 or 1			Cyclophosphamide (500 mg/m2 for day 1) + etoposide (100 mg/m2 on days 1–3) + cisplatin (100 mg/m2 on day 1) for 3 cycles, intravenous			37		
Rosell *et al*. [5]	Resectable NSCLC, free of metastases, Karnofsky index ≥ 60			Mitomycin (6 mg/m2 on day 1) + ifosfamide (3 g/m2 on days 1–3) + cisplatin (50 mg/m2 on days 1–3), intravenous			24		
Zhou *et al*. [6]	Resectable NSCLC, stage IIIA/B, 18 – 70 years, Karnofsky indetx ≥ 90, M0, N0/1/2			BAI (21)/MVP (68)/CAP (36)/EP (67)/VIP (30)/GP (30)/NP (32)/TP (10)/TN (30) for 2 cycles, intravenous and intraarterial			72 (12–132)		
Depierre *et al*. [7]	Resectable NSCLC, stage I (exclude T1N0), II, IIIA, ≤ 75 years, WHO performance status ≤ 2			Mitomycin (6 mg/m2 on day 1) + ifosfamide (1.5 g/m2 on days 1–3) + cisplatin (30 mg/m2 on days 1–3) for 2 cycles, intravenous			80		
Liao *et al*. [8]	Resectable NSCLC, stage I (exclude T1N0), II, IIIA, ≤ 75 years, Karnofsky index ≥ 80			MVP/MAP for 2 cycles (days 1, 8, and 15), intravenous			NR		
JCOG [9]	Resectable NSCLC, stage III, N2, M0, Zubrod perform status 0 or 1,< 76 years			Cisplatin (80 mg/m2 on day 1) + vindesine (3 mg/m2 on days 1 and 8), intravenous			74 (41–94)		
Gilligan *et al*. [10]	Resectable NSCLC, WHO performance status 0–2, M0			MVP (70)/MIP (41)/NP (216)/PC (2)/DC (69)/GP (130) for 3 cycles, intravenous			41 (30–58)		
Felip *et al*. [11]	Resectable NSCLC, stage IA (tumor size > 2 cm), IB, II, T3N1, ≥ 18 years, ECOG 0–2			Paclitaxel (200 mg/m2) + carboplatin for 3 cycles, intravenous			51		
Pisters *et al*. [12]	Resectable NSCLC, stage T2N0, T1–2N1, T3N0–1, ≥ 18 years, Zubrod perform status 0 or 1			Paclitaxel (225 mg/m2) + carboplatin for 3 cycles, intravenous			NR		
Scagliotti *et al*. [13]	Resectable NSCLC, stage I (exclude T1N0), II, IIIA, ≥ 18 years, Eastern Cooperative Oncology Group (ECOG) 0 or 1			Gemcitabine 1250 mg/m2 on days 1 and 8 every 21 days, and cisplatin 75 mg/m2 on day 1 for 3 cycles, intravenous			NR		
Chen *et al*. [14]	Resectable NSCLC, stage I (exclude T1N0), II, IIIA, < 75 years, Karnofsky index ≥ 80			Mitomycin (6 mg/m2) + cisplatin (80 mg/m2) + vindesine (2.5 mg/m2) on days 1, 8, and 15 for 1–2 cycles, intravenous			54 ± 49		

^a^Matching: 1, age; 2, sex; 3, ECOG performance status; 4, histological grade; 5, T stage; 6, N stage; 7, M stage; 8, histological type.

JCOG, Japan Clinical Oncology Group; NSCLC, non-small cell lung cancer; NAC, neoadjuvant chemotherapy; SA, surgery alone; NR, not reported; OS, overall survival; PFS,progression free survival; MVP, mitomycin + cisplatin + vinblastine; MIP, mitomycin + ifosfamide + cisplatin; BAI, bronchial artery infusion, CAP, cyclophosphomide + cisplatin + doxorubicin; VIP, cisplatin + ifosfamide + VP16; GP, gemcitabine + cisplatin; DC, carboplatin + docetaxel; PC, paclitaxel + carboplatin; NP, navelbine + cisplatin; TP, taxol + cisplatin; TN, taxol + navelbine; EP, vp16 + cisplatin.

### Methodological quality assessment

All of the selected articles had allocation concealment, blinding of observers and patients, and adequate sequence generation. With a median Jadad score of 3 (range, 2–5), the trials had relatively good methodological quality. Potential risk of bias lied in the facts that 6 trials did not report allocation concealment, 7 did not address loss of follow-up, and 4 did not report sample size calculation (Table 2).

**Table 2:** Quality assessment and risk of bias summary

Items	Roth et al. [4]	Rosell et al.[5]	Zhou et al. [6]	Depierre et al.[7]	Liao et al. [8]	JCOG [9]	Gilligan et al. [10]	Felip et al. [11]	Pisters et al. [12]	Scagliotti et al. [13]	Chen et al. [14]
Adequate sequence generation?	YES	YES	YES	YES	YES	YES	YES	YES	YES	YES	YES
Allocation concealment?	YES	YES	Unclear	YES	YES	Unclear	Unclear	YES	Unclear	Unclear	NR
Blinding (observer)?	NO	NO	NO	NO	NO	NO	NO	NO	NO	NO	NO
Blinding (patient)?	NO	NO	NO	NO	NO	NO	NO	NO	NO	NO	NO
Incomplete outcome data addressed?	NO	NO	NO	NO	NO	NO	YES	NO	NO	YES	NO
Postoperative protocol reported?	YES	YES	YES	YES	YES	Unclear	Unclear	YES	YES	YES	YES
Adequate report on loss to follow-up?	Unclear	Unclear	YES	Unclear	YES	NO	NO	Unclear	Unclear	YES	YES
Free of selective reporting?	YES	YES	YES	YES	YES	YES	YES	YES	YES	YES	YES
Free of other bias?	YES	YES	YES	YES	YES	YES	YES	YES	YES	YES	YES
Sample size calculation?	YES	NO	NO	YES	NO	YES	YES	YES	YES	YES	NO
Jadad score	5	3	2	4	4	4	2	3	3	2	2

JCOG, Japan Clinical Oncology Group.

### Primary outcomes

### NAC versus US in overall Survival (OS)

**Figure 2:**
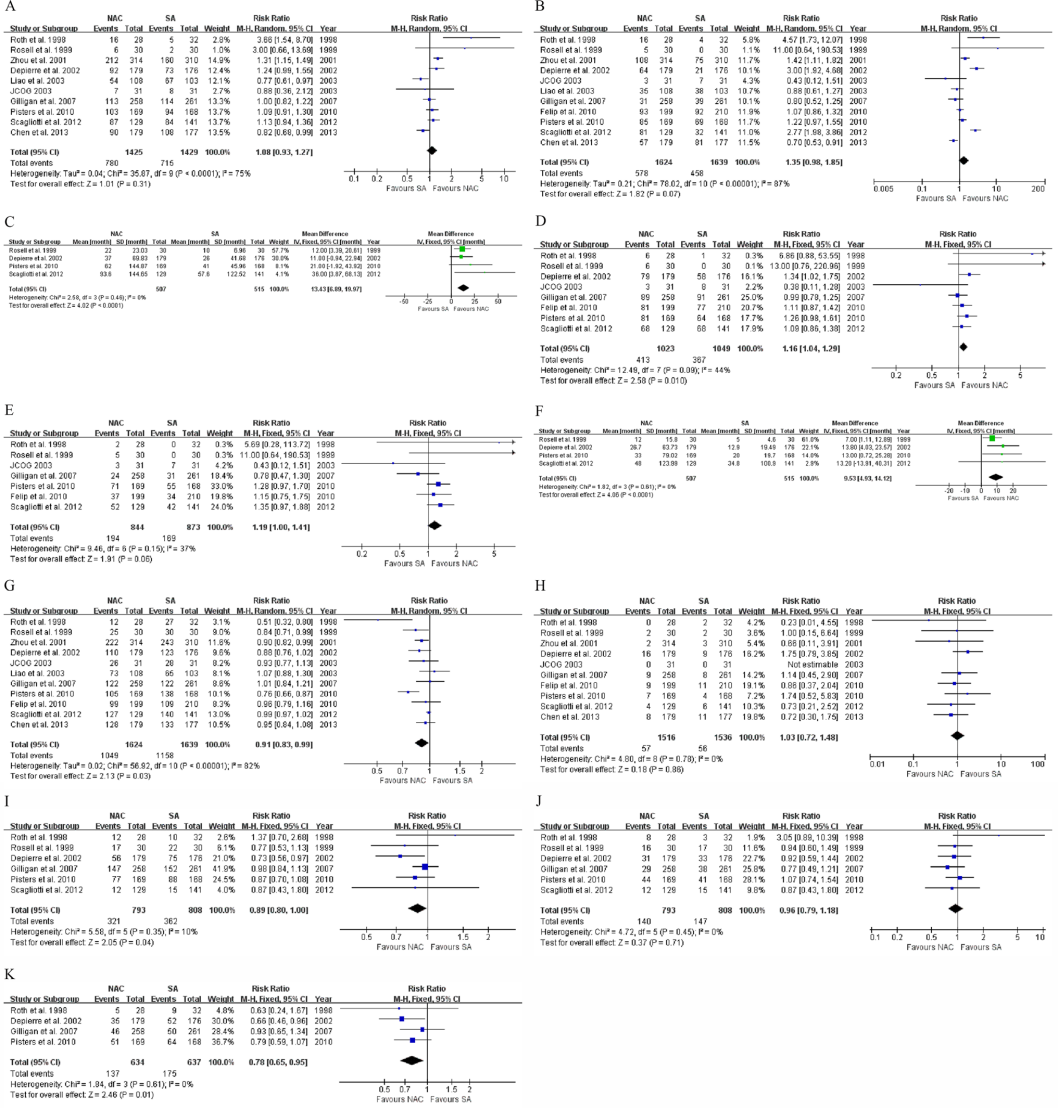
Forest plots of (**A**) 3-year overall survival, (**B**) 5-year overall survival, (**C**) pooled overall survival duration, (**D**) 3-year disease-free survival, (**E**) 5-year disease-free survival, (**F**) pooled disease-free survival duration, (**G**) total mortality, (**H**) perioperative mortality, (**I**) total recurrence, (**J**) local recurrence, and (**K**) distant metastasis when comparing NAC with US. NAC, neoadjuvant chemotherapy; US, upfront surgery; IV, inverse variance; M-H, Mantel-Haenszel; CI, confidence interval.

**Figure 3:**
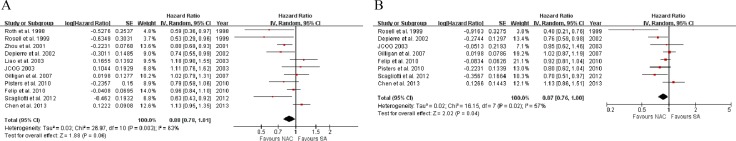
Forest plots of hazard ratio concerning overall survival (**A**) and disease-free survival (**B**) when comparing NAC with US. NAC, neoadjuvant chemotherapy; US, upfront surgery; IV, inverse variance; CI, confidence interval.

There was no significant difference in 3-year survival rates between the NAC and US groups (10 RCTs [8–14, 16–18], 54.7% *vs*. 50.0%, RR: 1.08, 95% CI: 0.93–1.27, *P* = 0.31, Figure 2A), while NAC tended to be associated with improved 5-year OS (all RCTs, 35.6% *vs*. 27.9%, RR: 1.35, 95% CI: 0.98–1.85, *P* = 0.07, Figure 2B). Both pooled-analyses had significant heterogeneity (χ^2^ = 35.87, *P <* 0.0001, *I*^2^ = 75%; χ^2^ = 70.61, *P* < 0.00001, *I*^2^ = 87%), so the random-effects model was chosen. Pooled survival duration based on 4 trials [9, 11, 16, 17] suggested a significant difference between the NAC and US arms (53.7 *vs*. 33.7 months, WMD: 13.43, 95% CI: 6.89–19.97, *P* < 0.0001, Figure 2C) using the fixed-effect model. The synthesized HR of NAC versus US was 0.88 (95% CI: 0.78–1.01, *P* = 0.06), based on the random-effects model due to significant heterogeneity (χ^2^ = 26.97, *P* = 0.003, *I*^2^ = 63%, Figure 3A).

### NAC versus US in disease-free survival (DFS)

Results for 3- and 5-year DFS were available in 8 [8, 9, 11, 13–17] and 7 [8, 9, 13–17] RCTs, respectively. With no significant heterogeneity, the fixed-effect model was applied in these analyses. Compared to US, NAC significantly contributed to a better 3-year DFS rate (40.4% *vs*. 35.0%, RR: 1.16, 95% CI: 1.04–1.29, *P* = 0.01, Figure 2D; RD: 0.07, 95% CI: 0.00 to 0.13, *P* = 0.04), and tended to be associated with a higher 5-year rate (23.0% *vs*. 19.4%, RR: 1.19, 95% CI: 1.00–1.41, *P* = 0.06, Figure 2E). Furthermore, pooled DFS duration based on 4 studies [9, 11, 16, 17] suggested a significant difference between the NAC and US groups (29.9 *vs*. 18.2 months, WMD: 9.53, 95% CI: 4.93–14.12, *P <* 0.0001, Figure 2F) using the fixed-effect model. The pooled HR of NAC versus US was 0.87 (95% CI: 0.76–1.00, *P* = 0.04), based on the random-effects model due to significant heterogeneity (χ^2^ = 16.15, *P* = 0.02, *I*^2^ = 57%, Figure 3B).

### NAC versus US in mortality

Results for total and perioperative mortalities were available in all and 10 [8–11, 13–18] RCTs, respectively. Due to significant heterogeneity (χ^2^ = 56.92, *P <* 0.00001, *I*^2^ = 82%), the random-effects model was chosen, and the total mortality at the end of follow-up of the US group was significantly higher than that of the NAC group (70.7% *vs*. 64.6%, RR: 0.91, 95% CI: 0.83–0.99, *P* = 0.03, Figure 2G; RD: –0.07, 95% CI: –0.13 to –0.02, *P* = 0.009). In terms of perioperative mortality, there was no significant difference between the NAC and US groups (3.8% *vs*. 3.7%, RR: 1.03, 95% CI: 0.72–1.48, *P* = 0.86, Figure 2H) using the fixed-effect model.

### NAC versus US in recurrence and metastasis

Applying the fixed-effect model due to insignificant heterogeneity, the NAC group had a significantly lower overall recurrence rate than the US group regarding postsurgical recurrence (6 trials [8, 9, 11, 14, 16, 17], 46.5% *vs*. 52.0%, RR: 0.89, 95% CI: 0.80–1.00, *P* = 0.04, Figure 2I; RD: –0.05, 95% CI: –0.11 to 0.00, *P* = 0.04). Based on the fixed-effect model due to insignificant heterogeneities, no significant difference was found in local recurrence rate between the NAC and US groups (6 trials [8, 9, 11, 14, 16, 17], 17.7% *vs*. 18.2%, RR: 0.96, 95% CI: 0.79–1.18, *P* = 0.71, Figure 2J), while there was a significant difference in distant metastasis incidence (4 RCTs [8, 11, 14, 16], 21.6% *vs*. 27.5%, RR: 0.78, 95% CI: 0.65–0.95, *P* = 0.01, Figure 2K; RD: –0.06, 95% CI: –0.11 to 0.01, *P* = 0.01).

### Secondary outcomes

### NAC versus US in tumor resection

**Figure 4:**
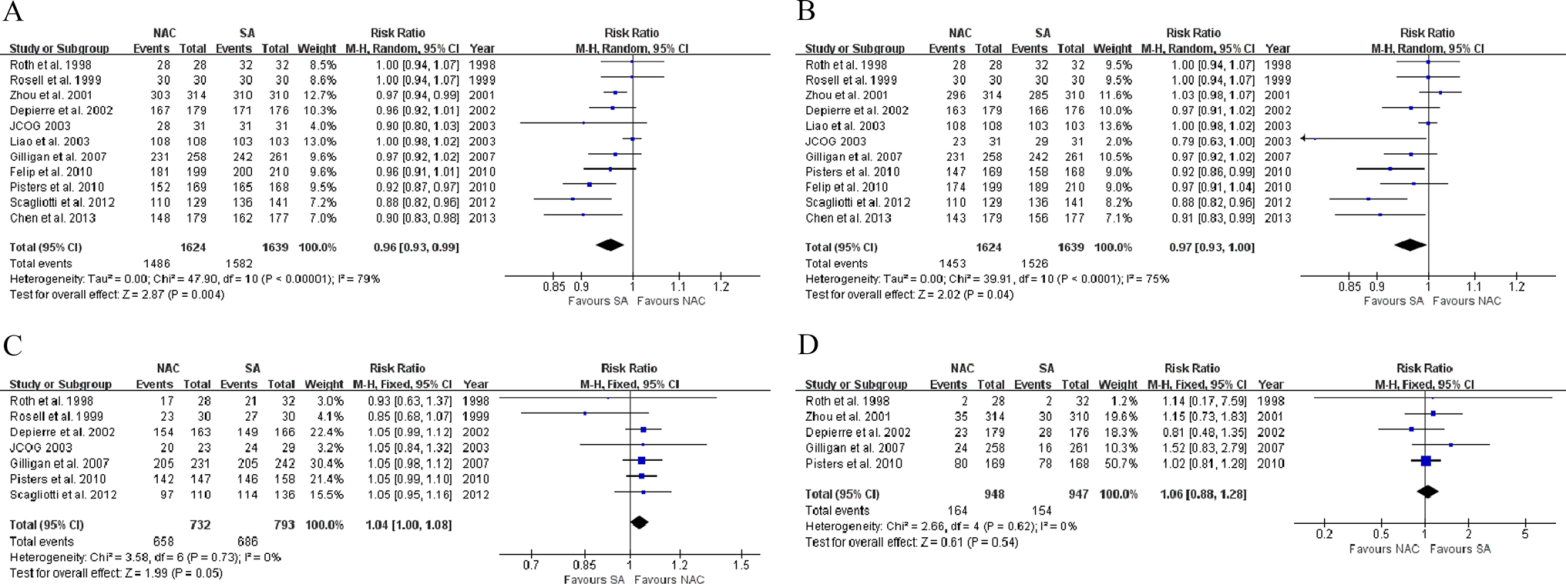
Forest plots of (**A**) surgery, (**B**) resection, (**C**) margin-negative resection among resected patients, and (D) postsurgical adverse events when comparing NAC with US. NAC, neoadjuvant chemotherapy; US, upfront surgery; M-H, Mantel-Haenszel; CI, confidence interval.

Based on all RCTs, NAC was significantly associated with lower surgery (91.5% *vs*. 96.5%, RR: 0.96, 95% CI: 0.93–0.99, *P* = 0.004, Figure 4A; RD: –0.04, 95% CI: –0.07 to –0.01, *P* = 0.003) and resection rates (89.5% *vs*. 93.1%, RR: 0.97, 95% CI: 0.93–1.00, *P* = 0.04, Figure 4B; RD: –0.03, 95% CI: –0.06 to 0.00, *P* = 0.03) compared to US, using the random-effects model due to significant heterogeneities (χ^2^ = 47.90, *P <* 0.00001, *I*^2^ = 79%; χ^2^ = 39.91, *P <* 0.0001, *I*^2^ = 75%). With insignificant heterogeneity, the fixed-effect model applied showed that among resected patients, NAC was associated with a higher R0 resection rate compared to US (7 RCTs [8, 9, 11, 13, 14, 16, 17], 89.9% *vs*. 86.5%, RR: 1.04, 95% CI: 1.00–1.08, *P* = 0.05, Figure 4C; RD: 0.03, 95% CI: 0.00 to 0.06, *P* = 0.04).

### NAC versus US in postoperative adverse events

Through the analysis of 5 RCTs [8, 10, 11, 14, 16], we found no significant difference in postoperative complication rate between the NAC and US groups (17.3% *vs*. 16.3%, RR: 1.06, 95% CI: 0.88–1.28, *P* = 0.54, Figure 4D), using the fixed-effect model.

### Subgroup analysis

In occident patients, the NAC group tended to have a higher 3-year OS rate (6 RCTs [8, 9, 11, 14, 16, 17], 52.6% *vs*. 46.0%, RR: 1.17, 95% CI: 0.99–1.37, *P* = 0.07, Supplementary Figure 1A), and significantly had a higher 5-year OS rate (7 RCTs [8, 9, 11, 14–17], 37.8% *vs*. 25.3%, RR: 1.78, 95% CI: 1.15–2.76, *P* = 0.01, Supplementary Figure 1B) than the US group using the random-effects model (χ^2^ = 10.99, *P* = 0.05, *I*^2^ = 55%; χ^2^ = 49.75, *P <* 0.00001, *I*^2^ = 88%). While in the orient subgroup, with the random-effects model applied (χ^2^ = 23.94, *P <* 0.0001, *I*^2^ = 87%; χ^2^ = 16.86, *P* = 0.0008, *I*^2^ = 82%), no significant differences were found between the NAC and US arms in 3-year (4 RCTs [10, 12, 13, 18], 57.4% *vs*. 55.2%, RR: 0.94, 95% CI: 0.67–1.31, *P* = 0.72, Supplementary Figure 1A) or 5-year (4 RCTs [10, 12, 13, 18], 32.1% *vs*. 32.4%, RR: 0.89, 95% CI: 0.57–1.39, *P* = 0.61, Supplementary Figure 1B) survival rate.

### Objective response to NAC

The objective response was reported in 10 RCTs [8–14, 16–18] (Supplementary Table 3), showing that 6.8% (110/1616) of patients had CR, and 45.3% (732/1616) had PR. The overall response rate (CR+PR) was 52.1% (842/1616). A total of 78 (4.8%) patients receiving NAC had PD.

### Safety analysis

According to the Common Toxicity Criteria of the National Cancer Institute, we studied the NAC-related adverse events in 8 RCTs [8, 10, 11, 13–17] (Supplementary Table 4). The overall toxicity rate was 58.3%. The most common NAC-related adverse effects were leucopenia (20.8%) and nausea/vomiting (10.6%), which were reported in all of the 8 investigated RCTs. Based on 3 studies [10, 11, 14], serious alopecia was observed in 7.3% of patients in the NAC group.

### Sensitivity analysis

Sensitivity analyses were performed for all the outcomes, yielding similar results or patterns (data not shown). Funnel plots (Supplementary Figure 2), Egger's tests (data not shown), and an exhaustive literature retrieval conferred a substantial confidence degree in our pooled results.

### Random-effects model-based results

If there was no heterogeneity, results were firstly pooled using the fixed-effect model, followed by using the random-effects model. For OS duration (Supplementary Figure 3A), DFS duration (Supplementary Figure 3B), 5-year DFS rate (Supplementary Figure 4B), perioperative mortality rate (Supplementary Figure 4C), local recurrence rate (Supplementary Figure 4E), distant metastasis rate (Supplementary Figure 4F), R0 resection rate among resected patients (Supplementary Figure 4G), and postsurgical adverse event rate (Supplementary Figure 4H), patterns and significances of results based on the random-effects model were consistent with those based on the fixed-effect model. However, based on the random-effects model, no significant differences were observed between the NAC and US arms regarding 3-year DFS rate (40.4% *vs*. 35.0%, RR: 1.15, 95% CI: 0.97–1.36, *P* = 0.11, Supplementary Figure 4A) or total recurrence rate (46.5% *vs*. 52.0%, RR: 0.89, 95% CI: 0.77–1.02, *P* = 0.09, Supplementary Figure 4D), although the patterns were consistent with those based on the fixed-effect model.

## DISCUSSION

Due to the fact that NSCLC could easily develop systemic dissemination, many patients have advanced disease at diagnosis and require systemic treatment, whereas surgery and radiotherapy being local treatment modalities play a minor role in systemic control [25]. NAC has been proved to be effective in other cancers, especially in breast cancer, against which it has already been increasingly used [26]. There are many advantages of NAC, including a better control of micro-metastasis and the potential to increase R0 resection rate through tumor shrinkage. However, the adverse events of chemotherapy potentially increasing postoperative morbidity and/or mortality rates and the delay of surgery might not be avoided. NAC in NSCLC has been studied and applied in the clinical setting since 1980s. It was reported to improve the survival compared to US [2, 27], but some researchers found confounding results [16]. Most of the published articles are needed to be treated with caution for their small sample size or nonrandomized designs, leaving the efficacy of NAC against NSCLC obscure.

This meta-analysis pooled data from 11 high-quality RCTs concerning NAC and US in non-metastatic NSCLC, which were selected based on strict eligibility criteria. Most of them are multicentric trials. Our pooled-analysis supports the efficacy of NAC in non-metastatic NSCLC. Some of the pooled results are inconsistent with those of the previous analyses [28]. There are many potentially-explanatory influential factors, including the discrepant percentages of men and squamous cell cancers, the difference in chemotherapy regimens, and the various intervals between randomization and surgery.

Some studies [8–10, 12, 16, 17] reported that NAC was effective and safe, and could significantly improve long-term survival. However, other trials [11, 13–15, 18] demonstrated no significant survival difference between the NAC and US groups. After pooling the data from the RCTs, we found improved survival in the NAC group compared to the US group. NAC was associated with over 10% reduced risk in death and recurrence. In the subgroup analysis according to ethnicity, occident patients receiving NAC had significantly higher 5-year survival rates than those undergoing US, and although with no significant difference, tended to have better 3-year survival rates, which is different from the results based on oriental participants, suggesting that occidental patients might have better responses to NAC. The difference could also be possibly explained by the difference in the chemotherapy regimens applied. Our study further showed that NAC was associated with significantly lower total mortality rate compared to the US group, especially in occident patients. However, the pooled perioperative mortality was comparable between the 2 groups. The observed ethnic differences could possibly be explained by the genetic background which needs further clarification.

NAC was associated with improved disease-free survival, total recurrence and distant metastasis rates while no difference was observed in the local recurrence rate. These might suggest that NAC is especially efficient in systemic and distant control, rather than locoregional control. To further achieve local control, radiotherapy might play a role, however it is not investigated in the included studies. Total mortality rate was lower in the NAC group compared to the US group at the end of follow-up, the duration of which was comparable between the two arms, while no difference in perioperative mortality rate was observed. This may suggest that NAC-associated toxicity does not increase short-term mortality risk, and that NAC provides survival benefit especially in the long term. Interestingly, while the opposite pattern is expected, NAC was associated with reduced surgery and resection rates, which could be partly due to the side effects of NAC and the changes in tissue such as fibrosis rendering surgery challenging. However, among resected patients, NAC was significantly associated with higher R0 resection rates. These might suggest that NAC could help to select the appropriate NSCLC candidates for whom curative resection is more likely and appropriate, and to rule out those with more aggressive tumor biology enabling the tumor to progress during neoadjuvant treatment. The promising improved R0 resection rates with NAC further supported the efficacy of NAC, and the fact that it could contribute to long-term survival in selected patients due to improved local control. No significant difference existed in postoperative complications, indicating NAC as a safe approach in terms of surgery.

In investigated studies, the overall response rate was about 50%, and approximately 5% of patients receiving NAC developed progressive disease. The response rate could be affected by various factors including chemotherapy regimen and administration route. In the JCOG trial [13], the rate was the lowest (25.8%). In Zhou *et al*.'s study [10], which had the highest rate, intra-arterial chemotherapy was performed for a significant proportion of the patients receiving NAC. Notably, the mitomycin/ifosfamide/cisplatin combination regimen in analyzed trials had response rates over 55%, while the rates in other trials applying different regimens were markedly lower. The NAC-related adverse event rate was 58.3%, and grade 3–4 toxicities (*e.g*., leukocytopenia and thrombopenia) were reported by most trials, which should be noteworthy. However, the toxicities did not result in higher rates of post-operative mortality, suggesting them as being well-manageable.

Compared with a comprehensive meta-analysis by the NSCLC Meta-analysis Collaborative Group [29] on preoperative chemotherapy for NSCLC published in 2014, our work uniquely focused on NAC versus US in non-metastatic NSCLC and provided results of subgroup analyses according to race. We also included a newly published trial [18] and two additional Chinese studies [10, 12], and had larger sample size. Compared with another previously published meta-analysis [28], our study included mere randomized phase III clinical trials and excluded some ineligible studies, so the results could be more convincing. Our analyses revealed that, NAC was especially effective in improving the long-term survival of occident patients with non-metastatic NSCLC. By the advent of more effective therapeutics more patients will benefit from treatment with fewer local recurrences, distant metastases, and NAC-related adverse events. Further in-depth investigation is needed.

This meta-analysis has some limitations, majorly reflected by the various regimens, administration courses and intervals between randomization and surgery, the absence of some 16 outcomes of interest in some trials, the occasional inter-trial heterogeneity, and the fact that not all parameters of interest were reported by all of the RCTs. More high-quality multicentric randomized trials with longer follow-up and larger sample sizes might be needed to further strengthen certain effects and to update the present findings with the advancement of regimens. Furthermore, results in the included trials were not separately reported for different age groups, tumor stages, histology groups, or surgical procedures, and the corresponding subgroup analyses were not possible. Nevertheless, the thorough literature retrieval, careful trial selection with only RCTs included, large sample size, and in-depth analyses of subgroups provided convincing evidence about the role of NAC in NSCLC.

In summary, NAC may provide better survival, reduced recurrence, and improved R0 resection rates among NSCLC patients who had surgery, especially in occident patients. Objective response rate may be an important advantage of NAC, and the adverse effects might be manageable.

## MATERIALS AND METHODS

### Literature search

We systematically searched MEDLINE, EMBASE, and the Cochrane Library electronic databases with the search terms “neo(-)adjuvant/pre(-)operative/pre(-)surgical chemotherapy”, “surgery/operation/resection”, and “lung/pulmonary/bronchial cancer/carcinoma/tumo(u)r/neoplasm/malignancy”. Reference lists of relevant publications and conference proceedings were also screened to ensure the comprehensiveness of the trial selection. No restrictions on language were applied during the retrieval.

### Inclusion and exclusion criteria

We only included clinical trials on pathologically diagnosed non-metastatic NSCLC patients (classified by the National Comprehensive Cancer Network (NCCN) [30]) who had NAC and surgery or surgery alone. Eligible RCTs comparing NAC with US enrolled individuals without age, sex and racial limitations, who were naive for chemo(radio)therapy, and who were in good condition to receive surgery, regardless of the chemotherapeutic regimen and dose, surgical procedure and tumor stage. The exclusion criteria were non-randomized studies, trials with only 1 arm receiving postoperative therapy, and those including patients with other pulmonary diseases (*e.g*., pneumonia and tuberculosis) unless separate results were reported. Studies were excluded from analysis if the retrieved paper was an earlier report of data updated in a subsequent publication which could cover all the information contained in the previous one.

### Outcomes of interest and definition

The 3- and 5-year survival rates, overall survival (OS), disease-free survival (DFS), total and perioperative mortalities, and recurrence were primary outcomes. Secondary outcomes included overall and R0 resection rates, postoperative complications, and NAC-related response and toxicity. Survival time was calculated from the start of randomization to death or the end of follow-up. Tumor down-staging effects were evaluated by comparing post-treatment stages to preoperative ones. The NAC-related pathological responses were classified into complete response (CR), partial response (PR), minor response (MR), stable disease (SD), and progressive disease (PD), based on the NCCN criteria [31].

### Literature quality assessment

Risk of bias was assessed for eligible literatures using the Cochrane Collaboration's tool and the Jadad scoring system, with trials scoring more than 2 as high-quality studies. The quality of included RCTs was also assessed according to the Consolidated Standards of Reporting Trials (CONSORT) statement [32]. The assessment was completed at the beginning of this analysis.

### Data extraction

The full texts of all relevant trials were reviewed separately by the 2 authors (X.N.Z. and L.H.). The publication information, inclusion and exclusion criteria, patients’ characteristics, and tumor and treatment information were initially extracted. For dichotomous and continuous outcomes, the data were recorded using case event and mean with standard deviation (SD), respectively. If the mean or SD was not reported or could not be calculated, then the median or range was applied for imputation according to data availability [33]. The hazard ratios (HRs) of OS and DFS were also extracted from the included studies.

### Statistical analysis

This study was conducted according to the PRISMA guidelines and the Cochrane Collaboration Guidelines [34] and based on ITT analysis. Results were pooled when reported by multiple trials using risk ratio (RR) for dichotomous data or weighted mean difference (WMD) for continuous results [35]. Risk difference (RD) was quantified in case of significant RR. For DFS and OS, HR was synthesized. The corresponding 95% confidence interval (CI) was calculated. The Mantel-Haenszel and inverse-variance methods were applied to analyze dichotomous and continuous data, respectively. The Higgins χ^2^ test was used to evaluate the heterogeneity and the inconsistency was quantified by the *I*^2^ value. Both the fixed-effect and the DerSimonian random-effects models were used if no heterogeneity existed (χ^2^ *P* > 0.100, *I*^2^ < 50%); otherwise, only the random-effects model was applied. Subgroup analyses were further conducted according to ethnicity. The funnel plot and the Egger's test [36] were applied to assess bias. Sensitivity analyses were conducted by excluding single trial. Data were managed and analyzed using the RevMan v. 5.3 and Stata software, with 2-sided *P* < 0.05 indicating statistical significance.

### Ethical

All procedures performed in studies involving human participants were in accordance with the ethical standards.

## SUPPLEMENTARY MATERIALS FIGURES AND TABLES



## Abbreviations

NSCLC: non-small cell lung cancer
NAC: neoadjuvant chemotherapy
RCT: randomized controlled trials
US: upfront surgery
OS: overall survival
DFS: disease free survival
PRISMA: Preferred Reporting Items for Systematic reviews and Meta-Analysis
ITT: intention-to-treat
CR: complete response
PR: partial response
MR: minor response
SD: stable disease
PD: progressive disease
NCCN: National Comprehensive Cancer Network
SD: standard deviation
HR: hazard ratio
RR: risk ratio
RD: risk difference
WMD: weighted mean difference
CI: confidence interval
