# Supplementary material for: Neoadjuvant chemotherapy followed by surgery versus upfront surgery in non-metastatic non-small cell lung cancer: systematic review and meta-analysis of randomized controlled trials
Source: Oncotarget. 2017 Aug 8;8(52):90327–37. doi: 10.18632/oncotarget.20044 (PMC5685753; doi:10.18632/oncotarget.20044)
Supplement: Supplementary file 1 [file oncotarget-08-90327-s001.pdf]

Neoadjuvant chemotherapy followed by surgery versus upfront surgery in non-metastatic non-small cell lung cancer: systematic review and meta-analysis of randomized controlled trials

SUPPLEMENTARY MATERIALS

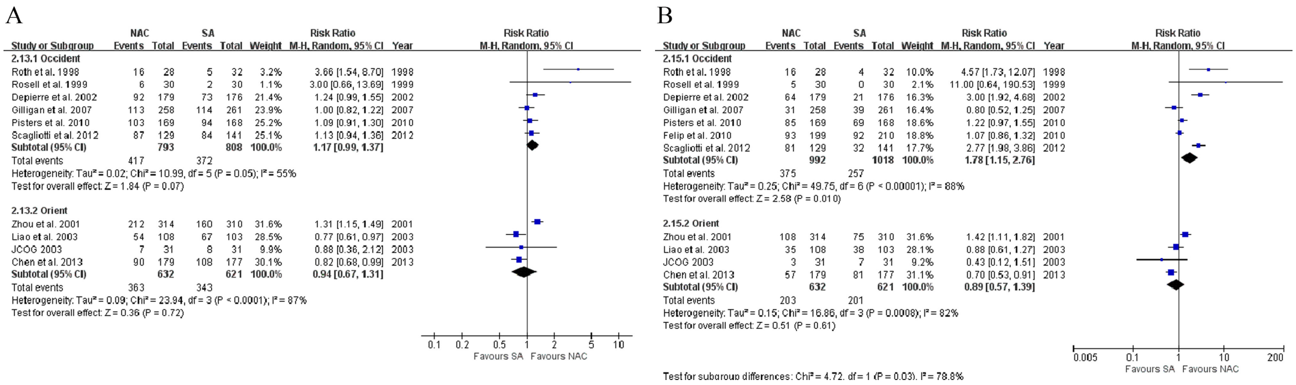

Supplementary Figure 1: Subgroup analysis of 3-year (**A**) and 5-year (**B**) overall survival rates according to ethnicity. NAC, neoadjuvant chemotherapy; US, upfront surgery; M-H, Mantel-Haenszel; CI, confidence interval.

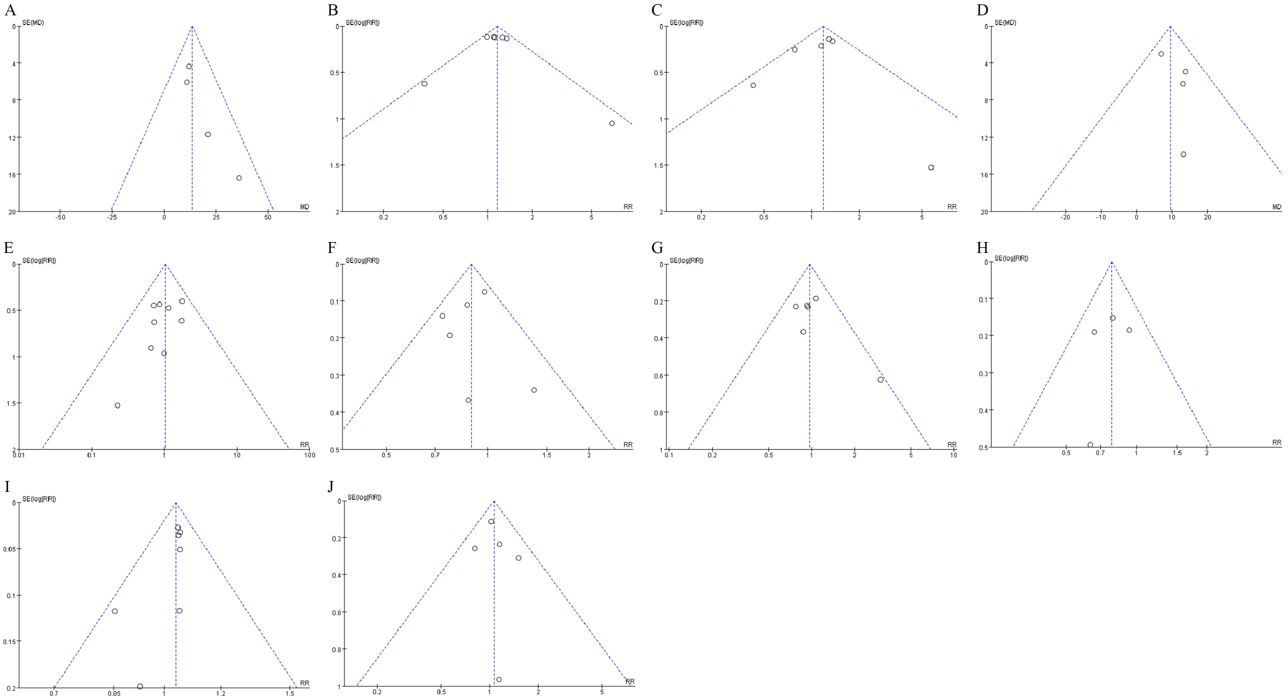

Supplementary Figure 2: Funnel plots of (**A**) overall survival duration, (**B**) 3-year disease free survival, (**C**) 5-year disease free survival, (**D**) disease-free survival, (**E**) perioperative mortality, (**F**) total recurrence, (G) local recurrence, (**H**) distant metastasis, (**I**) margin-negative resection among resected patients, and (**J**) postsurgical adverse events, all indicating insignificant bias. MD, mean difference; RR, risk ratio.

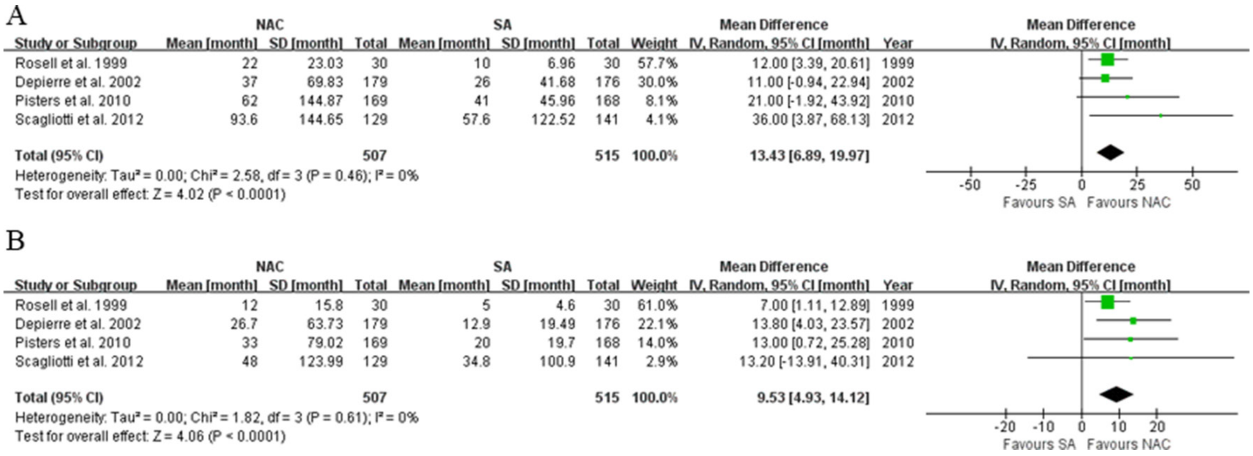

Supplementary Figure 3: Random-effects model-based forest plots of (**A**) pooled overall survival duration and (**B**) pooled disease-free survival duration when comparing NAC with US. NAC, neoadjuvant chemotherapy; US, upfront surgery; IV, inverse variance; CI, confidence interval.

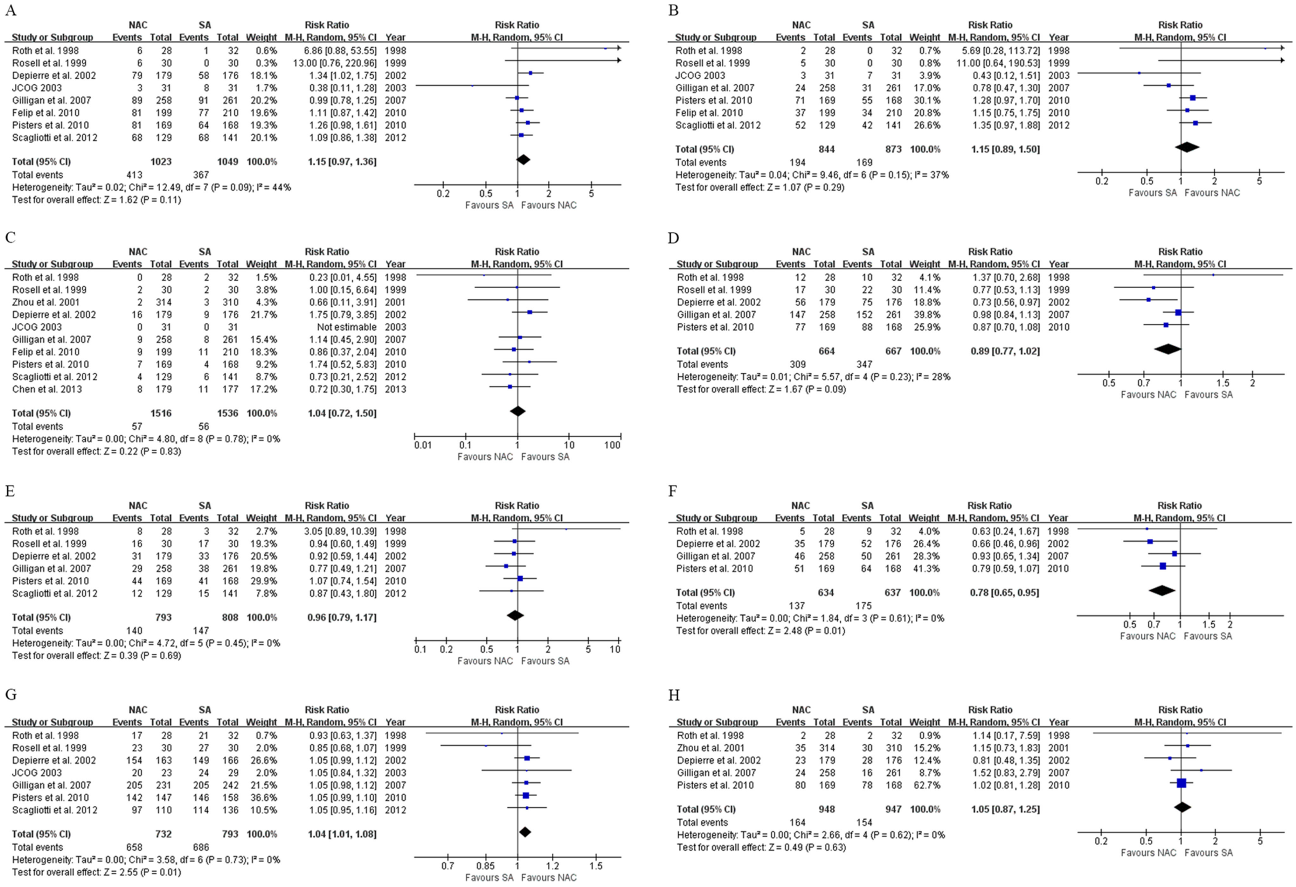

Supplementary Figure 4: Random-effects model-based forest plots of (**A**) 3-year disease-free survival, (**B**) 5-year disease-free survival, (**C**) perioperative mortality, (**D**) total recurrence, (**E**) local recurrence, (**F**) distant metastasis, (**G**) R0 resection among resected patients, and (**H**) postsurgical adverse events when comparing NAC with US. NAC, neoadjuvant chemotherapy; US, upfront surgery; M-H, Mantel-Haenszel; CI, confidence interval.

Supplementary Table 1: Patients' characteristics

Authors	Method	*n*	Age (year)	Sex (F/M)	ECOG score (0/1/2)	Histology (S/A/others)	T status (T1/T2/T3/T4)	cTNM (I/II/IIIa/IIIb)	Follow–up duration (months)
Roth *et al.* [4]	NAC	28	50 (36–67)	9/19	2/26/0	11/12/5	1/13/13/1	0/6/20/1	37
	SA	32	59 (40–72)	7/25	0/32/0	11/18/3	0/17/15/0	0/8/24/0	37
Rosell *et al.* [5]	NAC	30	60 (39–78)	1/29	NR	19/8/3	2/15/13/0	0/4/26/0	24
	SA	30	63 (43–72)	0/30	NR	23/6/1	1/14/15/0	0/9/21/0	19
Zhou *et al.* [6]	NAC	314	55 ± 10 (35–70)	74/240	NR	164/106/44	0/25/269/20	0/0/289/25	72 (12–132)
	SA	310	56 ± 11 (36–70)	71/239	NR	157/101/52	0/40/255/15	0/0/291/19	72 (12–132)
Depierre *et al.* [7]	NAC	179	60 (37–73)	12/167	115/60/4	129/30/20	7/143/29/0	62/25/92/0	80
	SA	176	61 (37–75)	11/165	113/60/3	134/30/12	4/143/29/0	69/32/75/0	80
Liao *et al.* [8]	NAC	108	61 (39–75)	23/85	NR	56/35/17	NR	47/24/37/0	NR
	SA	103	64 (24–75)	22/81	NR	43/38/22	NR	52/23/28/0	NR
JCOG [9]	NAC	31	59 (32–74)	11/20	31/0	7/21/3	2/25/3/1	0/0/31/0	74 (41–94)
	SA	31	61 (45–74)	10/21	31/0	8/20/3	11/17/3/0	0/0/31/0	74 (41–94)
Gilligan *et al.* [10]	NAC	258	62 (37–77)	71/186	139/114/5	131/67/60	NR	165/72/19/2	41
	SA	261	63 (25–79)	72/188	144/113/4	125/71/65	NR	154/91/15/1	41
Felip *et al.* [11]	NAC	199	65 (35–80)	24/175	88/108/1	107/57/35	20/156/22/1	148/46/4/1	51
	SA	210	64 (36–89)	26/184	102/105/3	105/71/34	21/159/30/0	154/52/4/0	51
Pisters *et al.* [12]	NAC	169	65 (38–83)	36/64	66/34/69	34/31/41	NR	NR	64
	SA	168	64 (35–82)	32/68	63/37/68	42/33/48	NR	NR	64
Scagliotti *et al.* [13]	NAC	129	61 (38–76)	29/100	96/33/0	48/43/38	NR	56/67/6/0	40 (0–102)
	SA	141	63 (38–80)	16/125	99/42/0	63/42/36	NR	76/61/3/1	31 (0–102)
Chen *et al.* [14]	NAC	179	61 (34–75)	37/132	NR	79/62/28	NR	61/42/66/0	54 ± 49
	SA	177	62 (24–76)	41/127	NR	60/72/36	NR	78/34/56/0	54 ± 49

All values are presented as n or mean ± SD or median (range).
JCOG, Japan Clinical Oncology Group; F, female; M, male; NR, not reported; S, squamous cell carcinoma; A, adenocarcinoma.

Supplementary Table 2: Eligibility criteria for patient inclusion

Authors/Trial acronym	Symptoms and signs	Endoscopy/ Pathology	Imaging signs	Laboratory studies	Severe comorbidities	Previous/ Other therapy	Other malignancies
Roth et al. [4]	NR	YES	YES	YES	NO	NO	NR
Rosell et al. [5]	YES	YES	YES	YES	NO	NR	NR
Zhou et al. [6]	YES	YES	YES	NR	NO	NO	NO
Depierre et al. [7]	NR	YES	YES	YES	NR	NO	NR
Liao et al. [8]	YES	YES	YES	NR	NO	NR	NO
JCOG [9]	NR	YES	YES	YES	NO	NO	NO
Gilligan et al. [10]	NR	YES	YES	NR	NO	NO	NO
Felip et al. [11]	YES	YES	YES	YES	NO	NO	NO
Pisters et al. [12]	YES	YES	YES	YES	NO	NR	NR
Scagliotti et al. [13]	NR	YES	NR	NR	NO	NO	NO
Chen et al. [14]	YES	YES	YES	NR	NO	NO	NO

JCOG, Japan Clinical Oncology Group; NR, not reported.

Supplementary Table 3: Chemotherapeutic response according to the RECIST criteria

Authors	*n*	Complete response	Partial response	Minor response	Stable disease	Progressive disease
Roth et al. [4]	30	1 (3.3%)	8 (26.7%)	8 (26.7%)	3 (10.0%)	4 (13.3%)
Rosell et al. [5]	30	2 (6.7%)	16 (53.3%)	NR	11 (36.7%)	1 (3.3%)
Zhou et al. [6]	314	50 (15.9%)	181 (57.6%)	NR	NR	NR
Depierre et al. [7]	179	19 (10.9%)	95 (53.1%)	NR	47 (26.3%)	10 (5.6%)
Liao et al. [8]	108	1 (0.9%)	53 (49.1%)	4 (3.7%)	NR	0
JCOG [9]	31	0	8 (25.8%)	NR	12 (38.7%)	9 (29.0%)
Gilligan et al. [10]	258	9 (3.5%)	111 (43.0%)	NR	69 (26.7%)	15 (5.8%)
Felip et al. [11]	199	18 (9.0%)	88 (44.2%)	NR	63 (31.7%)	11 (5.5%)
Pisters et al. [12]	169	5 (3.0%)	64 (37.9%)	NR	73 (43.2%)	11 (6.5%)
Scagliotti et al. [13]	129	4 (3.1%)	40 (31.5%)	NR	55 (43.3%)	7 (5.5%)
Chen et al. [14]	169	1 (0.6%)	68 (40.2%)	NR	10 (5.9%)	10 (5.9%)
Total response	1616	110 (6.8%)	732 (45.3%)	12 (0.7%)	343 (21.2%)	78 (4.8%)

JCOG, Japan Clinical Oncology Group; NR, not reported.

Supplementary Table 4: NAC-associated acute toxicity

Category	Roth *et al.* [4]	Zhou*et al.* [6]	Depierre *et al.* [7]	JCOG [9]	Gilligan *et al.* [10]	Felip *et al.* [11]	Pisters *et al.* [12]	Scagliotti *et al.* [13]	Total (%)
n (NAC)	28	314	179	31	258	193	169	129	1446
Leukopenia	22 (78.6%)	23 (7.3%)	6 (3.4%)	10 (32.3%)	NR	62 (32.1%)	81 (47.9%)	41 (31.8%)	301 (20.8%)
Thrombopenia	1 (3.6%)	19 (6.1%)	1 (0.6%)	NR	NR	21 (10.9%)	1 (0.6%)	16 (12.4%)	61 (4.2%)
Anemia	NR	9 (2.9%)	NR	4 (12.9%)	NR	76 (39.4%)	0	3 (2.3%)	95 (6.6%)
Nausea/vomiting	10 (35.7%)	26 (8.3%)	26 (14.5%)	4 (12.9%)	31 (12.02%)	38 (19.7%)	6 (3.6%)	6 (4.7%)	153 (10.6%)
Alopecia	NR	15 (4.8%)	26 (14.5%)	NR	40 (15.50%)	NR	NR	NR	84 (5.8%)
Lethargy/fatigue	NR	NR	NR	NR	43 (16.67%)	57 (29.5%)	86 (50.9%)	2 (1.6%)	192 (13.3%)
Diarrhea	7 (25%)	NR	NR	NR	NR	14 (7.3%)	NR	NR	24 (1.7%)
Hypomagneseia	10 (35.7%)	NR	NR	NR	NR	NR	NR	NR	10 (0.7%)
Total (%)	50 (7.7%)	92 (14.1%)	59 (9.1%)	18 (2.8%)	114 (17.48%)	268 (38.9%)	174 (26.7%)	68 (10.4%)	843 (58.3%)

NAC, neoadjuvant chemotherapy; JCOG, Japan Clinical Oncology Group; NR, not reported.
